# Auto-Transplantation of Teeth: A Descriptive Cross-Sectional Study of Knowledge and Attitude

**DOI:** 10.7759/cureus.48614

**Published:** 2023-11-10

**Authors:** Lena S Elbadawi, Abdulrahman Al Farhah

**Affiliations:** 1 Department of Periodontics, King Abdulaziz University, Jeddah, SAU; 2 Department of General Dentistry, King Abdulaziz University, Jeddah, SAU

**Keywords:** questionnaire, tooth replacement, cross-sectional study, periodontology, auto-transplantation

## Abstract

Aim: This study aimed to evaluate the knowledge and attitude of dental interns regarding tooth auto-transplantation and to determine the need for additional education on this topic among dental interns.

Methods: Ethical approval was obtained for the study. A self-administered questionnaire was designed, validated, and distributed to dental interns from various dental schools in the Western Province of Saudi Arabia. The questionnaire covered demographics, knowledge assessment, and attitude. The data were analyzed using chi-square and Fisher's exact tests, with gender as an independent variable.

Results: A total of 215 dental interns participated, with a response rate of 44.1%. Only 61.4% were familiar with teeth auto-transplantation. Dental interns displayed knowledge gaps and misconceptions concerning tooth auto-transplantation, with varying levels of understanding. The study revealed gender-related differences in knowledge and attitudes. Notably, a substantial portion of participants relied on social media and the internet for information about auto-transplantation.

Conclusion: The study identified significant knowledge gaps and misconceptions among dental interns regarding tooth auto-transplantation. To address these issues and promote a proper understanding of this treatment modality, it is crucial to integrate comprehensive education on auto-transplantation into undergraduate dental curricula. This will help bridge the knowledge-practice gap and dispel misconceptions surrounding this valuable procedure. Future research should consider larger sample sizes from private dental schools and explore the impact of improved education on attitudes and practices regarding tooth auto-transplantation among dental professionals.

## Introduction

Tooth auto-transplantation involves the surgical transfer of a tooth from one extraction site to another or to a prepared socket within the same individual [1,2]. This treatment modality has gained prominence as a viable option for replacing hopeless teeth due to its capacity to restore functionality akin to natural teeth [1]. Recently, its resurgence in popularity can be attributed to a deeper understanding of the periodontal ligament healing process. Tooth auto-transplantation has proven to be a valuable approach in cases of tooth loss resulting from trauma, caries, periodontitis, endodontic disorders, tooth impaction, agenesis, and other dental conditions [2].

Successful auto-transplantation of teeth, in contrast to osseointegrated dental implants, ensures a healthy periodontium, continued eruption, preservation of alveolar bone volume and interdental papilla, and the ability to respond to orthodontic or physiological adjustments [2-5]. One noteworthy advantage of auto-transplantation over dental implants is its applicability to children and adolescents who often experience tooth loss due to trauma [2]. As a result, auto-transplanted teeth perform similarly and could even surpass dental implants in terms of durability and long-term prognosis [6,7]. Nevertheless, complications, such as inflammatory and replacement root resorption, ankylosis, pulp necrosis, and limited periodontal healing, may arise [7]. All things considered, auto-transplantation is considered a safe and effective procedure, particularly for pediatric patients with developing dentitions, for whom alternative surgical interventions may not be as effective, especially given that recent evidence has demonstrated third molar development in patients as young as 12 years of age [1,8].

Moreover, the benefits extend beyond dental health, offering improvements in patients' appearance, arch form, dentofacial development, masticatory function, speech, and arch integrity [9]. Numerous studies have demonstrated its high success and survival rates [1,4-6,10,11]. In comparison to other treatment modalities like implants, prosthetic restorations, and orthodontic space closure, the adoption of total dental auto-transplantation remains relatively limited [9]. This may be attributed to misconceptions and a lack of awareness, as evidenced by previous studies [2,12]. Reports suggest that dental auto-transplantation is not commonly practiced in hospitals or taught to dental students [12]. Additionally, there is a persistent lack of awareness and misconception surrounding dental auto-transplantation [13].

The literature shows that there is a dearth of research assessing the level of knowledge and misconceptions about tooth auto-transplantation among dental interns or recently graduated dental students. Consequently, the limited awareness and knowledge assessment may contribute to the underutilization of auto-transplantation as a valid treatment modality. As such, the aim of our study is to evaluate the knowledge and attitude of dental interns regarding tooth auto-transplantation and to determine the need for additional education on this topic among dental interns.

## Materials and methods

The study received approval from the Research Ethics Committee at King Abdulaziz University Faculty of Dentistry (REC-KAUFD) under approval number #44-49739, dated 22/11/2022. The target sample for this study consisted of dental interns from multiple dental schools in the Western Province of Saudi Arabia.

An online self-administered questionnaire in English was created using Google Forms and distributed to the intended sample via official invitation letters sent by email to the respective internship office of each of the targeted dental schools. To safeguard the anonymity of all participants, no names or identifying information were collected, in accordance with the ethical principles outlined in the Declaration of Helsinki. The consent statement underscored participants' rights to withdraw from the study at any point without incurring any penalties and was obtained electronically from all participants before filling out the questionnaire. The questionnaire was composed of three sections: demographics, knowledge assessment, and attitude. The demographics section featured three multiple-choice questions aimed at capturing the characteristics of the respondents including gender, college name, and familiarity with auto-transplantation. Given that all participants were interns, collecting age information was deemed unnecessary. The knowledge assessment section comprised five multiple-response questions, which were designed to gauge the participants' fundamental understanding of the procedure and to identify any potential misconceptions, which was achieved by the inclusion of multiple false answers under each question. High-quality evidence was utilized to reference both the correct and incorrect answers [1,2,14]. The attitude section consisted of five Likert-scale questions intended to measure the participants' attitude regarding auto-transplantation as a treatment modality, with answers to each question ranging from strongly agree to strongly disagree. All questions and their answers can be seen in the Results section.

The questionnaire underwent a comprehensive validation process, including both content and face validation, with the input of expert academicians and feedback from a representative 10% of the target sample. The validation feedback received was highly satisfactory, indicating no need for revisions.

To determine the appropriate sample size, we employed an online sample size calculator (Raosoft). Our calculations were based on a 5% margin of error and a 95% confidence interval within a population of 487 dental interns from the targeted dental schools. This computation led to a target sample size of 215 dental interns.

Data coding and analysis were conducted using IBM SPSS (International Business Machines Corporation's Statistical Package for the Social Sciences for Windows, version 26.0; IBM Corp, Armonk, NY). Given the nature of our survey, only participants who answered yes to being familiar with auto-transplantation were included in the reporting and analysis. Descriptive statistics for all questionnaire items were summarized as frequencies and percentages. To assess the items related to knowledge and attitude, we employed chi-square and Fisher's exact tests, as appropriate. For analytical convenience when testing the attitude section, we combined "highly agree" and "agree" into one group, and "disagree" and "highly disagree" into another group. Statistical significance was defined as a p-value of <0.05.

## Results

A total of 215 dental interns from various dental schools in the western region of Saudi Arabia participated in this study, with an overall response rate of 44.1%. Among the respondents, 132 of them (61.4%) reported being familiar with auto-transplantation, with the majority being female (n = 74, 56.1%). A summary of the respondents' characteristics is presented in Table 1.

**Table 1:** Descriptive statistics of the sample characteristics (n = 132)

Variable		n	%
Gender	Female	74	56.1
	Male	58	43.9
College	King Abdulaziz University	103	78
	Batterjee College	11	8.30
	Ibn-Sina National College	7	5.30
	Taibah University	2	1.50
	Umm Al-Qura University	5	3.80
	Vision College	4	3

Questions of the knowledge and attitude section items were assessed using chi-square and Fisher's exact tests, with gender used as an independent variable. Descriptive statistics for the questions and their corresponding responses are outlined in Tables 2, 3.

**Table 2:** Descriptive statistics of the knowledge section (n = 132) ^‡^Multiple-response question, participants who answered with “yes”. Bold entries represent correct answers.

Question		n	%
How did you get familiar with auto-transplantation?	Internet sources	55	41.7
	Scientific conferences	22	16.7
	Social media	82	62.1
	Undergraduate lectures	60	45.5
	Workshops	2	1.5
What is considered a potential indication of tooth auto-transplantation?^‡^	Movement of an impacted or severely ectopic tooth	25	19
	Premature loss of deciduous tooth	7	5.3
	Premature loss of permanent tooth	71	54
	Replacement of a permanent first molar with a poor prognosis	100	76
	Replacement of traumatized teeth with poor prognosis	44	33
	Transplantation of a badly decayed tooth	14	10.6
	Transplantation of a necrotic tooth	17	12.9
What is considered a contraindication of teeth auto-transplantation?^‡^	Deciduous dentition	109	83
	Growing patient	49	37.1
	Incomplete root formation	56	42.4
	Non-compliant patient	65	49
	Permanent dentition	13	9.8
	Transplantation of a tooth into an infected site	95	72
	Transplantation of the periodontally affected tooth with attachment loss	40	30
What are the prognostic factors that affect teeth auto-transplantation success?^‡^	Alveolar bone level	95	72
	Developmental stage of the tooth	76	58
	Patient’s dental hygiene	89	67
	Postoperative stabilization	82	62
	Surgical technique	78	59
What are the common possible complications that can occur after teeth auto-transplantation?^‡^	Ankylosis	119	90
	Calcification of the root canal	37	28
	Internal root resorption	52	39.4
	Lack of or limited periodontal healing	46	35
	Lack of periodontal stability	72	54.5
	Postoperative infection	55	42

**Table 3:** Descriptive statistics of the attitude section (n = 132)

Statement		n	%
Teeth auto-transplantation is commonly underused as a treatment option	Strongly agree	31	23.5
	Agree	57	43.2
	Neutral	28	21.2
	Disagree	14	10.6
	Strongly disagree	2	1.5
I believe that teeth auto-transplantation has a high success rate	Strongly agree	10	7.6
	Agree	59	44.7
	Neutral	58	43.9
	Disagree	5	3.8
	Strongly disagree	0	0
I believe that majority of dental specialists implement teeth auto-transplantation in their practice	Strongly agree	3	2.3
	Agree	25	18.9
	Neutral	43	32.6
	Disagree	46	34.8
	Strongly disagree	15	11.4
Teeth auto-transplantation should be part of the undergraduate dental curriculum	Strongly agree	32	24.2
	Agree	55	41.7
	Neutral	32	24.2
	Disagree	8	6.1
	Strongly disagree	5	3.8
I'm interested in expanding my knowledge about teeth auto-transplantation	Strongly agree	64	48.5
	Agree	51	38.6
	Neutral	16	12.1
	Disagree	0	0
	Strongly disagree	1	0.8

When gender was used as an independent variable, seven statistically significant associations were identified. More males reported learning about teeth auto-transplantation from social media (p < 0.05). Likewise, more males believed that incomplete root formation should be considered a potential contraindication for teeth auto-transplantation, while more females considered permanent dentition a contraindication (p < 0.05). More males also believed that postoperative stabilization, surgical technique, and the developmental stage of the tooth significantly influence the success of auto-transplantation (p < 0.05). Finally, more females concurred that auto-transplantation should be integrated into the undergraduate curriculum (p < 0.05).

## Discussion

In this cross-sectional, questionnaire-based study, our primary aim was to assess the knowledge and attitude of dental interns concerning tooth auto-transplantation. A total of 215 dental interns consented to participation in the survey, and it was observed that 64% of them reported being familiar with the concept of tooth auto-transplantation, and thus answered the three sections of our questionnaire.

The knowledge section of our survey consisted of five questions, of which four covered potential indications, contraindications, prognostic factors, and potential complications associated with tooth auto-transplantation. When it comes to identifying the indications for auto-transplantation, there was a discernible trend with a majority of participants selecting the correct answer. Incorrect answers were chosen by only 12.9% of the participants or fewer. In contrast, the question about contraindications presented a more varied response, with participants selecting multiple incorrect answers nearly as much as they selected the correct ones. Although certain incorrect answers showed significant gender associations, these associations did not yield any substantial insights. However, when we questioned participants about prognostic factors influencing the success of auto-transplantation, a different pattern emerged. All the correct answers were selected by our participants by a majority of 57.6% or more. Furthermore, three out of the five correct answers showed significant associations with males, suggesting a better understanding of the prognostic factors among this group. This could be attributed to the potential for greater clinical exposure to auto-transplantation procedures among males, as suggested in previous studies [15]. Finally, when participants were asked about possible complications associated with auto-transplantation, there was no clear consensus among the responses, except for the choice "ankylosis," which was answered correctly by over 90% of participants. This finding aligns with previous literature and underscores the need for improved education and awareness regarding tooth auto-transplantation [13].

Overall, with the exception of the question concerning prognostic factors, our data reveal a deficiency in knowledge about tooth auto-transplantation as a treatment modality. There are prevalent misconceptions regarding its indications, contraindications, and potential complications. These findings are consistent with prior research in the field [13,15,16]. This is even more evident as the majority of our respondents stated that they have learned about auto-transplantation from social media, which is not a reliable source of information and thus could be a contributing factor to the widespread misconception. Originally, our intention was to compare the knowledge variations between governmental dental schools and private dental schools. However, due to the uneven distribution of respondents, with the majority coming from governmental schools, a statistically valid comparison was not feasible. We recommend that future research includes larger sample sizes from private dental schools to better evaluate potential variations in knowledge between graduates of these two sectors.

Shifting the focus to perceptions and attitudes toward tooth auto-transplantation, the majority of participants expressed the view that auto-transplantation is underutilized as a treatment option. However, only half believed that it is associated with a high success rate, and a minority suggested that auto-transplantation should be integrated into most dental specialties. These findings are consistent with a study conducted by Elizabeth et al., which found that 26.5% and 63.2% of targeted dentists reported that auto-transplantation is seldom or never practiced, respectively. Additionally, they found that 95.6% had never participated in an auto-transplantation procedure, and 40.6% were unsure of the prognosis associated with this procedure [15]. Another study by Al-Khanati et al., primarily including oral and maxillofacial surgeons and periodontists, revealed that over 70% of these professionals did not consider auto-transplantation when developing treatment plans, with 43.2% suggesting that there are better alternatives available [16]. These findings highlight potential misconceptions regarding the prognosis and reported outcomes of tooth auto-transplantation.

It is noteworthy that our sample consisted of dental interns, yet the results align with the findings of Elizabeth and Al-Khanati, which shed light on the fact that even as dentists progress from internship to practice and specialization, their attitudes and practices regarding auto-transplantation may not significantly change [15,16]. This underscores the pressing need to enrich undergraduate dental curricula with comprehensive coverage of auto-tooth transplantation-related topics, including its practice and documented success rates. The fact that a majority of our participants believe that auto-transplantation should be incorporated into the undergraduate curriculum underscores this need. Similarly, a significant number of participants expressed interest in expanding their knowledge about auto-transplantation, echoing the findings of Elizabeth et al., where over 95% of practicing dentists expressed a strong desire for more knowledge on the subject [15].

Interestingly, our study identified a statistically significant association between female participants and the belief that auto-transplantation should be a part of the undergraduate curriculum. This may be attributed to the observation that males tend to report greater exposure to auto-transplantation clinical procedures, while females express a higher need for exposure to these topics as part of their undergraduate education [15]. Nonetheless, it is essential to acknowledge the multiple shortcomings that this study has run through. The predominantly governmental school-based sample, with limited representation from private institutions, combined with the limited sample size, may restrict the generalizability of our findings. Additionally, the cross-sectional design only provides a snapshot of dental interns' knowledge and attitudes at one point in time, and longitudinal studies could offer more insights into changes over time. Despite these limitations, our study underscores the need for enhanced education and awareness surrounding tooth auto-transplantation, particularly as it relates to undergraduate dental curricula, in order to not only bridge the gap between knowledge and practice in the field of dentistry but to bust all the widespread myths and misconception regarding this treatment modality. 

## Conclusions

In conclusion, our study uncovers significant gaps in the knowledge and perceptions of dental interns regarding tooth auto-transplantation. Although a considerable number of participants are acquainted with the topic, it is concerning that their primary sources of information include social media and the internet. These findings underscore the necessity of integrating comprehensive education on this subject into undergraduate dental curricula while underscoring the need for heightened awareness and practical experience among dental professionals.

## Appendices

**Table 4:** Questionnaire used

Question	Answer
Age	(Open ended)
Gender	Male
	Female
College	AL-Batterjee College
	College of Dental Medicine at Umm Al-Qura University
	College of Dentistry at Taibah University
	College of Dentistry at Taif University
	Faculty of Dentistry at King Abdul-Aziz University
	Ibn-Sina National College for Medical Studies
	Vision College for Dentistry
Are you familiar with the term teeth auto-transplantation?	Yes
	No
How did you know about teeth auto-transplantation?	Internet sources (articles, search engines, etc.…)
	Lectures from curriculum
	Scientific conferences
	Social media
	Workshops
From the below options, what is considered a potential indication of tooth auto-transplantation? *(you can choose more than one answer)	Premature loss of permanent tooth (caries, trauma, iatrogenic damage)
	Premature loss of deciduous tooth (caries, trauma, iatrogenic damage)
	Replacement of a permanent first molar with a poor prognosis
	Transplantation of a badly decayed tooth
	Replacement of traumatized teeth with poor prognosis
	Movement of an impacted or severely ectopic tooth
	Transplantation of a necrotic tooth
From the below options, what is considered a contraindication of teeth auto-transplantation? *(you can choose more than one answer)	Deciduous dentition
	Permanent dentition
	Transplantation of a tooth into an infected site
	Growing patient
	Transplantation of periodontally affected tooth with attachment loss
	Non-compliant patient
	Incomplete root formation
From the below options, what are the prognostic factors that affect teeth auto-transplantation success? *(you can choose more than one answer)	Developmental stage of the tooth
	Alveolar bone level
	Surgical technique
	Postoperative stabilization
	Patient's dental hygiene
From the below options, what are the common possible complications that can occur after teeth auto-transplantation? *(you can choose more than one answer)	Ankylosis
	Internal root resorption
	Lack of or limited periodontal healing
	Postoperative infection
	Calcification of the root canal
	Lack of periodontal stability
Teeth auto-transplantation is commonly underused as a treatment option	Strongly agree
	Agree
	Neutral
	Disagree
	Strongly disagree
I believe that teeth auto-transplantation has a high success rate	Strongly agree
	Agree
	Neutral
	Disagree
	Strongly disagree
I believe that majority of dental specialists implement teeth auto-transplantation in their practice	Strongly agree
	Agree
	Neutral
	Disagree
	Strongly disagree
Teeth auto-transplantation should be part of the undergraduate dental curriculum	Strongly agree
	Agree
	Neutral
	Disagree
	Strongly disagree
I'm interested in expanding my knowledge about teeth auto-transplantation	Strongly agree
	Agree
	Neutral
	Disagree
	Strongly disagree
